# Fatty Acid-Treated Induced Pluripotent Stem Cell-Derived Human Cardiomyocytes Exhibit Adult Cardiomyocyte-Like Energy Metabolism Phenotypes

**DOI:** 10.3390/cells8091095

**Published:** 2019-09-17

**Authors:** Yuichi Horikoshi, Yasheng Yan, Maia Terashvili, Clive Wells, Hisako Horikoshi, Satoshi Fujita, Zeljko J. Bosnjak, Xiaowen Bai

**Affiliations:** 1 Department of Emergency Medicine, Asahikawa Medical University, Asahikawa, Hokkaido 078-8510, Japan; yhorikoshi-11@outlook.com (Y.H.); sfujita@asahikawa-med.ac.jp (S.F.); 2 Department of Anesthesiology, Medical College of Wisconsin, Milwaukee, WI 53226, USA; hishiha@juntendo.ac.jp; 3 Department of Cell Biology, Neuroscience & Anatomy, Medical College of Wisconsin, Milwaukee, WI 53226, USA; yashengyan@mcw.edu; 4 Department of Physiology, Medical College of Wisconsin, Milwaukee, WI 53226, USA; mterashv@mcw.edu (M.T.); zbosnjak@mcw.edu (Z.J.B.); 5 Department of Microbiology and Immunology, Medical College of Wisconsin, Milwaukee, WI 53226, USA; 6 Department of Plastic and Reconstructive Surgery, Juntendo University School of Medicine, Tokyo 113-8421, Japan; 7 Department of Medicine, Medical College of Wisconsin, Milwaukee, WI 53226, USA

**Keywords:** induced pluripotent stem cells, cardiomyocytes, maturation, metabolism

## Abstract

Human induced pluripotent stem cell (iPSC)-derived cardiomyocytes (CMs) (iPSC-CMs) are a promising cell source for myocardial regeneration, disease modeling and drug assessment. However, iPSC-CMs exhibit immature fetal CM-like characteristics that are different from adult CMs in several aspects, including cellular structure and metabolism. As an example, glycolysis is a major energy source for immature CMs. As CMs mature, the mitochondrial oxidative capacity increases, with fatty acid β-oxidation becoming a key energy source to meet the heart’s high energy demand. The immaturity of iPSC-CMs thereby limits their applications. The aim of this study was to investigate whether the energy substrate fatty acid-treated iPSC-CMs exhibit adult CM-like metabolic properties. After 20 days of differentiation from human iPSCs, iPSC-CMs were sequentially cultured with CM purification medium (lactate+/glucose-) for 7 days and maturation medium (fatty acids+/glucose-) for 3–7 days by mimicking the adult CM’s preference of utilizing fatty acids as a major metabolic substrate. The purity and maturity of iPSC-CMs were characterized via the analysis of: (1) Expression of CM-specific markers (e.g., troponin T, and sodium and potassium channels) using RT-qPCR, Western blot or immunofluorescence staining and electron microscopy imaging; and (2) cell energy metabolic profiles using the XF96 Extracellular Flux Analyzer. iPSCs-CMs (98% purity) cultured in maturation medium exhibited enhanced elongation, increased mitochondrial numbers with more aligned Z-lines, and increased expression of matured CM-related genes, suggesting that fatty acid-contained medium promotes iPSC-CMs to undergo maturation. In addition, the oxygen consumption rate (OCR) linked to basal respiration, ATP production, and maximal respiration and spare respiratory capacity (representing mitochondrial function) was increased in matured iPSC-CMs. Mature iPSC-CMs also displayed a larger change in basal and maximum respirations due to the utilization of exogenous fatty acids (palmitate) compared with non-matured control iPSC-CMs. Etomoxir (a carnitine palmitoyltransferase 1 inhibitor) but not 2-deoxyglucose (an inhibitor of glycolysis) abolished the palmitate pretreatment-mediated OCR increases in mature iPSC-CMs. Collectively, our data demonstrate for the first time that fatty acid treatment promotes metabolic maturation of iPSC-CMs (as evidenced by enhanced mitochondrial oxidative function and strong capacity of utilizing fatty acids as energy source). These matured iPSC-CMs might be a promising human CM source for broad biomedical application.

## 1. Introduction

Despite advances in medical technology and treatment, heart disease remains the leading cause of mortality worldwide partially due to the lack of appropriate human cardiomyocyte (CM) sources for repairing injured hearts, studying disease mechanisms, and screening drugs for efficacy/toxicity. For example, myocardial infarction is characterized by CM death following restricted blood flow [1]. As adult CMs have extremely limited proliferation capacity, injured heart function is progressively worsened, leading to severe long-term consequences such as arrhythmia, congestive heart failure, or even death. Although a broad array of treatment options is available, conventional management of heart failure generally does not successfully replace lost CM mass with new contractile cells [2,3,4,5,6]. In addition, there has been a long list of high-profile drugs that have been withdrawn from the market due to their off-target and clinical cardiac toxicities even after years of laboratory and preclinical testing. These drug withdrawals are partly attributable to inadequate screening using less than ideal animal models and non-CM cell lines (e.g., human embryonic kidney or Chinese hamster ovary cell lines), resulting in a tremendous burden on the healthcare system [7,8]. Human pluripotent stem cell (iPSC)-derived CMs (iPSC-CMs) are an ideal solution to these challenges by providing unlimited human CMs for myocardial regeneration, drug screening, and disease modeling [9,10,11].

iPSCs can be reprogrammed from many somatic cell sources (e.g., keratinocytes, renal epithelial cells in the urine, fibroblasts, or peripheral blood cells). Like embryonic stem cells, iPSCs have unlimited proliferation capacity and the ability to differentiate into many types of somatic cells [12,13,14]. With the rapid development and improvement of CM differentiation and enrichment techniques, highly purified contracting CMs can be obtained from iPSCs in culture [15,16,17,18,19]. However, mounting evidence demonstrates that iPSC-CMs exhibit immature fetal CM-like characteristics that are different from adult CMs in several aspects (e.g., cellular structure and metabolism), thereby limiting their aforementioned applications. Glycolysis is a major energy source of immature CMs to facilitate cells in a more proliferative state [20,21,22]. As CMs mature, mitochondrial oxidative capacity increases, with fatty acid β-oxidation becoming a key energy source (80%) to meet the heart’s high energy demand [23,24]. Immature iPSC-CMs may not be able to fully recapitulate the physiological and pathological changes occurring in the patient’s heart in response to stress and drugs. Additionally, immature iPSC-CMs may cause arrhythmia after transplantation into injured hearts for the purpose of myocardial regeneration.

Recent studies have shown that glucose is a negative regulator of the maturation of hESC-CMs [25], and that fatty acid-contained CM maturation medium enhanced the maturation of human iPSC-CMs in several aspects such as improved sarcomere organization, increased CM maturation-related gene expression, and enhanced contractility of iPSC-CMs [26]. Since an extraordinarily high energy demand is critical for the contractile function of adult CMs, it is important to have a better understanding of the energy metabolism-related properties in these matured iPSC-CMs to better utilize these matured human cells in heart repair, disease modeling and drug screening. Thus, the aim of this study was to investigate whether these fatty acid-mediated matured iPSC-CMs exhibit adult CM-like metabolic properties by analysis of glycolysis and mitochondrial oxidative function, and the ability of utilizing different exogenous energy (e.g., fatty acids and glucose) sources.

## 2. Materials and Methods

### 2.1. Human iPSC Culture and Expansion

**Figure 1:**
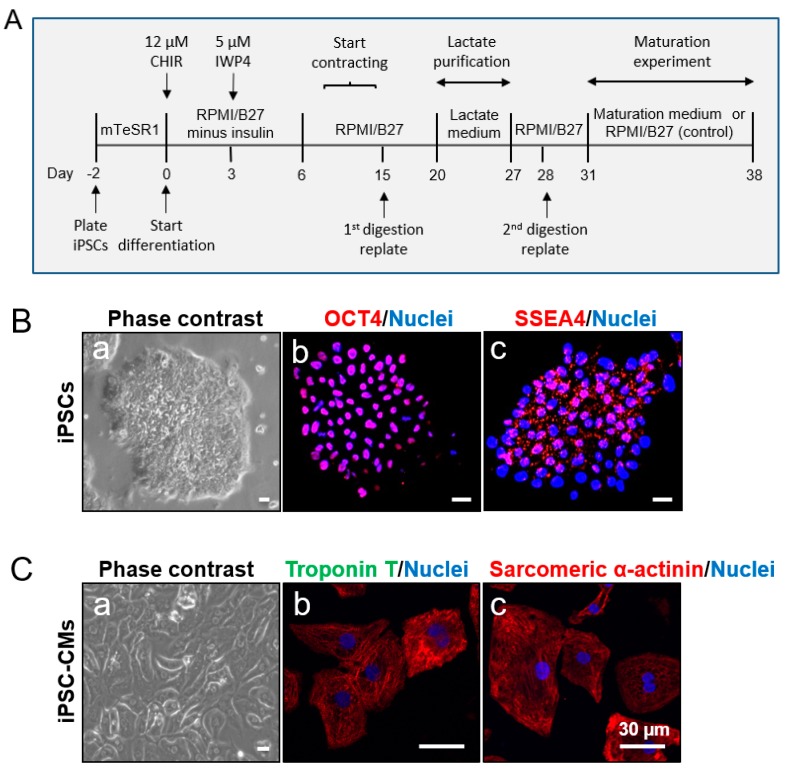
Characterization of human induced pluripotent stem cells (iPSCs) and iPSC-derived cardiomyocytes (iPSC-CMs). (**A**) Schematic depicting the procedure for the generation of cardiomyocytes from iPSCs by temporal modulation of Wnt signaling, purification, and maturation of iPSC-CMs. Note: mTeSR1 and Roswell Park Memorial Institute *(RMPI):* cell culture medium; B27: culture medium supplement; CHIR-99021: highly selective inhibitor of glycogen synthase kinase 3 (GSK-3); and IWP4: inhibitor of Wnt/β-catenin signaling. (**B**) Characterization of cultured 1013 iPSCs. Phase contrast image shows that iPSCs grow as colonies (a). Confocal fluorescent images indicate that iPSCs express pluripotent stem cell-specific markers octamer-binding transcription factor (OCT4) (red) (b), and stage-specific embryonic antigen-4 (SSEA4, red) (c). Blue are cell nuclei stained with Hoechst 33342. Scale bar = 50 μm. (**C**) Characterization of the differentiated cardiomyocytes (1013 iPSC-derived CMs). iPSC-CMs (day 20) grew as a monolayer (a) and expressed cardiomyocyte-specific markers troponin T (green) (b) and sarcomeric α-actinin (red) (c). Blue are cell nuclei. Scale bar = 30 µm.

**Figure 2:**
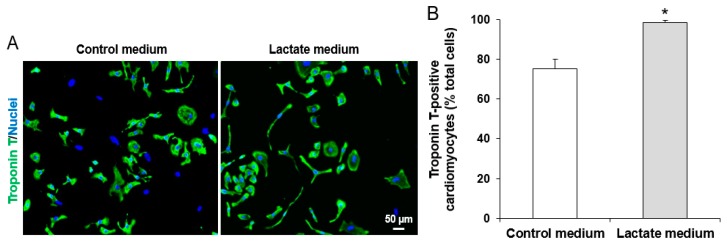
Lactate purification of 1013 iPSC-derived CMs. (**A**) The fluorescent images of iPSC-CMs (day 31) with or without treatment of lactate-contained purification medium (no glucose) for 7 days to eliminate non-cardiomyocytes. Blue are cell nuclei stained with Hoechst 33342 and green are troponin T signals. In the purified cell culture, almost all cells with blue nuclei expressed troponin T. Scale bar = 50 µm. (**B**) The purification of iPSC-CMs increased from 75% to 98% after culturing in lactate medium. Data are presented as mean ± SEM, *n* = 4 * *p* < 0.05 vs. control medium.

**Figure 3:**
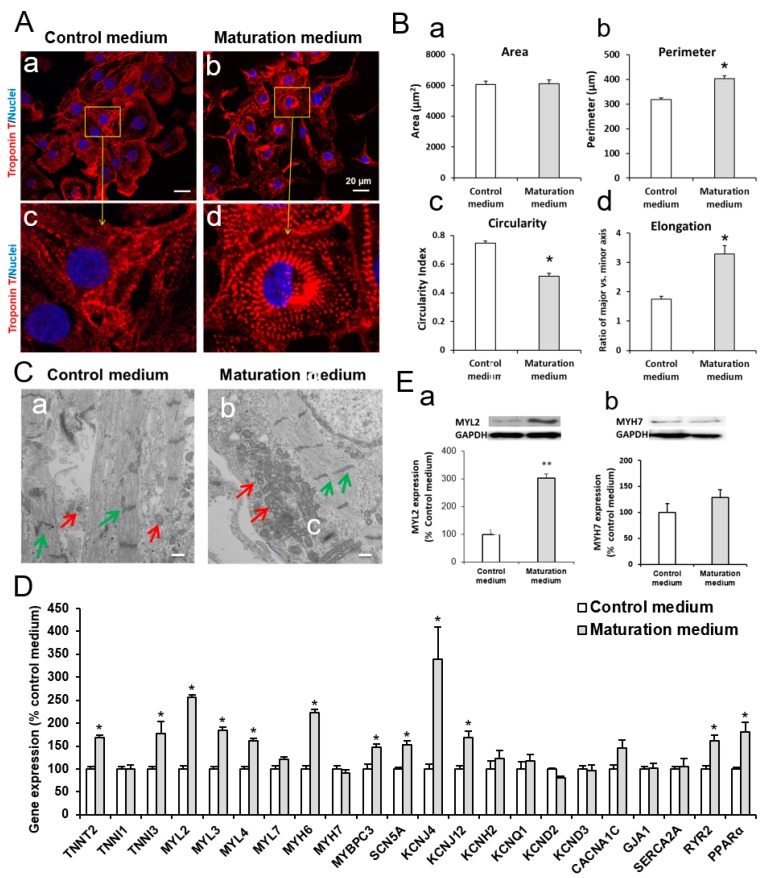
The effect of fatty acid-contained cardiomyocyte maturation medium (no glucose) on the maturation of 1013 iPSC-derived CMs. (**A**) Representative immunofluorescent images of iPSC-CMs (day 34) cultured with control culture medium (a) and maturation medium for 7 days (b). A-c and A-d are the magnified images marked by yellow rectangles in A-a and A-b, respectively. Scale bar = 20 µm. (**B**) Analysis of cell area (a), perimeter (b), circularity (c), and elongation (d) of iPSC-CMs using ImageJ software. *n* = 50–64 * *p* < 0.05 vs. control medium. (**C**) Representative electron microscopy images of iPSC-CMs (day 38) treated with control medium (a) and maturation medium (b) for 7 days. Scale bar = 500 nm. Maturation medium-treated iPSC-CMs showed well-aligned visible Z-lines and increased mitochondria within the cells compared with the cells cultured in control medium. Z-lines and mitochondrial are indicated by green and red arrows, respectively. (**D**) Real time PCR analysis of the effect of maturation medium on the cardiomyocyte maturation-related gene expression. *n* = 4 * *p* < 0.05 vs. control medium. (iPSC-CMs day 38) (**E**) Western blot analysis of the effect of maturation medium on MYL2 (a) and MYH7 (b) protein expression in iPSC-CMs (day 38). *n* = 4, ** *p* < 0.01, vs. control medium. Note: TNNI: troponin I; TNNT: troponin T; MYL: myosin light chain; MYH: myosin heavy chain; MYBPC: myosin binding protein C; SCN5A: sodium voltage-gated alpha subunit 5; KCNJ: potassium inwardly rectifying channel subunit J; KCNH: potassium voltage-gated channel subfamily H; KCNQ: potassium voltage-gated channel subfamily Q; KCND: potassium voltage-gated channel, subfamily D; CACNA1C: calcium voltage-gated channel subunit alpha1 C; GJA1: gap junction protein, alpha 1; SERCA2A: sarcoplasmic/endoplasmic reticulum Ca^2+^ATPase 2a; RYR2: cardiac ryanodine receptor; PPARα: peroxisome proliferator-activator receptor alpha.

**Figure 4:**
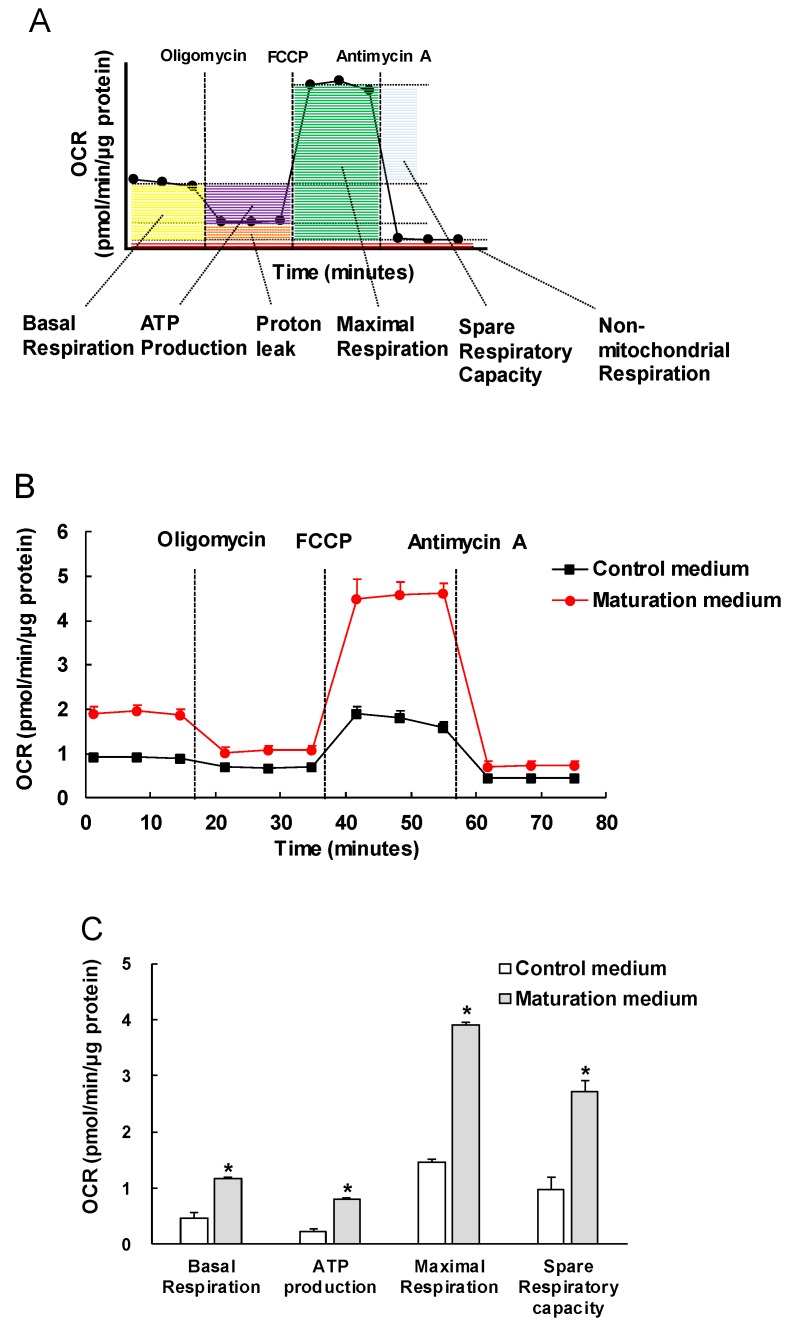
The effect of maturation medium on mitochondrial respiratory capacity of 1013 iPSC-derived CMs. (**A**) The diagram depicts the trace of oxygen consumption rate (OCR) on cells after sequentially administration of 10 µM oligomycin (ATP synthase inhibitor), 2 µM FCCP (uncoupler of oxidative phosphorylation in mitochondria), and 10 µM antimycin A (electron transport chain blocker) to the culture. The profiles of fundamental parameters of mitochondrial function measured are basal respiration, ATP production, maximal respiration and spare respiratory capacity that were marked with different color in the schematic. (**B**) Representative two OCR traces of iPSC-CMs (day 34) treated with either control medium or maturation medium for 3 days, respectively, in response to oligomycin, FCCP, and antimycin A. (**C**) OCR parameters representing mitochondrial function in maturation medium-treated iPSC-CMs were significantly higher. *n* = 4, * *p* < 0.05 vs. control medium.

**Figure 5:**
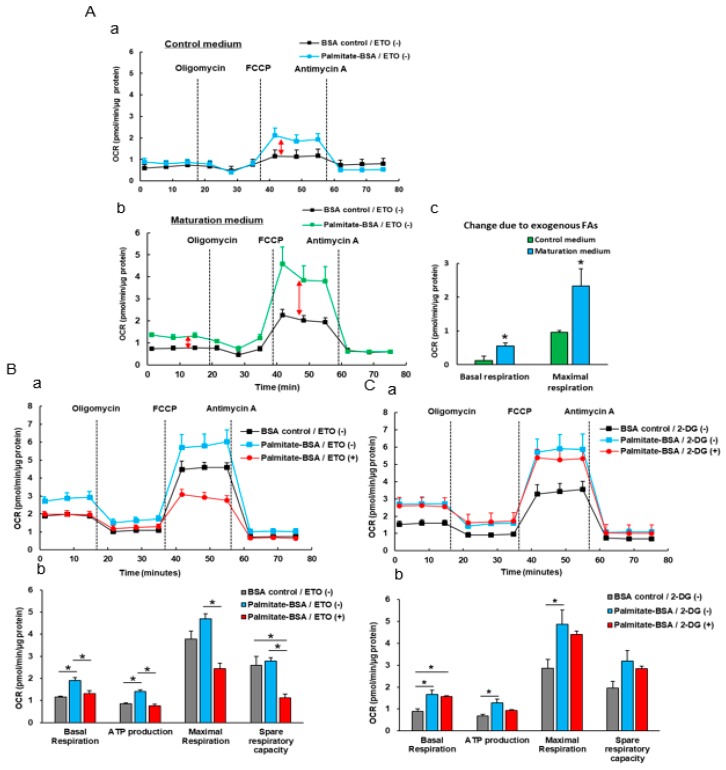
The effect of exogenous fatty acids (palmitate) pre-treatment on the OCR of 1013 iPSC-derived CMs (day 34) cultured with either control medium or maturation medium. (**A**) The changed basal and maximum OCR in both control and matured iPSC-CMs in response to exogenous palmitate. iPSC-CMs were pretreated with bovine serum albumin (BSA; as control), palmitate, or etomoxir (ETO, a specific irreversible inhibitor of carnitine palmitoyltransferase 1 inhibitor). Basal and maximum respiration capacity was significantly increased in the matured iPSC-CMs compared to control cells due to the utilization of palmitate. Pretreatment with palmitate-BSA (blue) resulted in the increased basal and maximal respiration in non-matured iPSC-CMs compared with BSA alone treatment group (black) (a). Matured iPSC-CMs exhibit the greater increase in both basal respiration (4.4 fold higher vs. non-matured iPSC-CMs) and maximal respiration (2.5 fold higher vs. non-matured iPSC-CMs) in response to palmitate-BSA (blue) (b and c), indicating the stronger ability to oxidize exogenously added palmitate in the matured iPSC-CMs. *n* = 4 * *p* < 0.05. (**B**) The effect of ETO on the OCR of matured iPSC-CMs pre-treated with BSA or palmitate. Representative OCR traces of iPSC-CMs pre-treated with BSA or palmitate together with or without ETO in response to oligomycin, FCCP, and antimycin A (a). ETO completely inhibited the palmitate pretreatment-mediated increases of mitochondrial respiration in matured iPSC-CMs (b). *n* = 4 * *p* < 0.05. (**C**) The effect of 2-deoxyglucose (2-DG) on the matured iPSC-CMs that were pre-treated with BSA or palmitate. Representative OCR traces of iPSC-CMs pre-treated with BSA or palmitate together with or without 2-DG (glucose analogue, a competitive glycolytic inhibitor) in response to oligomycin, FCCP, and antimycin A (a). 2-DG did not attenuate the palmitate-mediated increase of OCR in matured iPSC-CMs (b), suggesting that the palmitate-mediated increased OCR in palmitate-pretreated iPSC-CMs is resulted from the fatty acid effect. *n* = 4 * *p* < 0.05.

**Figure 6:**
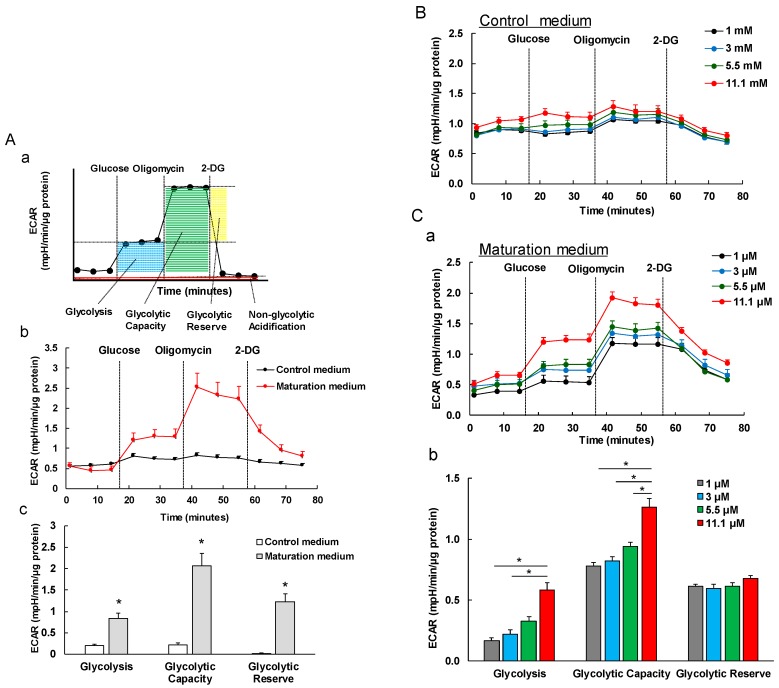
The effect of maturation medium on glycolytic function of iPSC-CMs (day 34) via analysis of the extracellular acidification rate (ECAR) of 1013 iPSC-derived CMs. (**A**-a) This diagram depicts (1) the representative traces of ECAR on the cells after sequentially adding 5.5 µM glucose, 10 µM oligomycin, and 25 mM 2-DG to the culture and (2) the profiles of key parameters of glycolytic function are glycolysis (blue), glycolytic capacity (green), glycolytic reserve (yellow), and non-glycolytic acidification (red). (**A**-b) Representative two ECAR traces of iPSC-CMs treated with control and maturation medium for 3 days, respectively. (**A**-c) The quantified ECAR shows that maturation medium increased glycolysis, glycolytic capacity, and glycolytic reserve of iPSC-CMs, suggesting that matured iPSC-CMs have a higher capacity to switch to glucose utilization when fatty acid oxidation is compromised. *n* = 4, * *p* < 0.05 vs. control medium. (**B**) The effects of different concentrations of glucose on glycolytic function of the control medium-treated iPSC-CMs. There is no significant difference in glycolytic function for each glucose concentration. (**C**-a) Representative traces of ECAR of matured iPSC-CMs in response to various glucose concentrations. (**C**-b) The glycolysis and glycolytic capacity exhibited the response to the glucose in a dose-dependent manner. *n* = 4, * *p* < 0.05.

**Figure 7:**
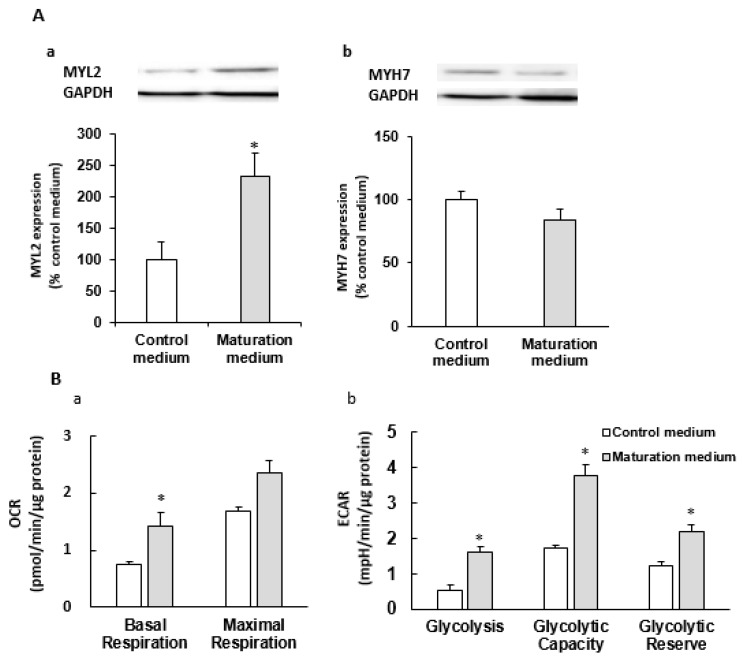
The effect of the maturation medium on protein expression, and metabolism of H3083 iPSC-derived CMs (**A**) Western blot analysis of the effect of maturation medium treatment for 7 days on the expression of MYL2 and MYH7 proteins in iPSC-CMs (day 38). *n* = 4 * *p* < 0.05, vs. control medium. (**B**) The effect of maturation medium on OCR and ECAR of iPSC-CMs (day 34). * *p* < 0.05 vs. control medium.

Two iPSC lines (1013 and H3083) were used in this study. The data in Figure 1, Figure 2, Figure 3, Figure 4, Figure 5, Figure 6 and Figure 7 were generated using the 1013 and H3083 iPSC lines, respectively. 1013 iPSC line was generated from dermal fibroblasts isolated from a 36-year-old healthy donor (male) in the laboratory of Dr. Douglas Melton (Department of Stem Cell and Regenerative Biology, Harvard University). H3083 iPSC line was reprogrammed from a 59-year-old healthy donor (male)-derived peripheral blood mononuclear cells in the laboratory of Dr. Jean Jieun Kim (Baylor College of Medicine). iPSCs were cultured in Matrigel (Corning Inc., Corning, NY, USA)-coated petri dishes with mTeSR1 medium (feeder-free cell culture medium for iPSCs; STEMCELL Technologies, Vancouver, BC, Canada) supplemented with 1% penicillin/streptomycin (Thermo Fisher Scientific, Waltham, MA, USA) (Table 1) in a hypoxic incubator (4% O_2_, 5% CO_2_) at 37 °C as described previously [18,19]. The culture media was changed daily. iPSCs were gently dissociated and passaged at a ratio of 1:6 using Versene (Thermo Fisher Scientific) when they reached about 70–80% confluence as follows. The culture medium was aspirated, and iPSCs were washed with Dulbecco’s phosphate-buffered saline (DPBS, Thermo Fisher Scientific) twice, and dissociated with Versene for 7–10 min at 37 °C, followed by adding Dulbecco’s modified Eagle medium/nutrient mixture F-12 (DMEM/F-12, Thermo Fisher Scientific). The dissociated cell suspension was carefully transferred into a 15 mL tube and centrifuged at 154× *g* for 5 min. The supernatants were discarded and the cell pellets were resuspended with fresh mTeSR1 medium and plated on Matrigel-coated dishes for culture as described above.

### 2.2. Generation of CMs from iPSCs

Prior to the initiation of CM differentiation from iPSCs, iPSCs were dissociated into single cells by treatment of 3 mL Accutase (Innovative Cell Technologies, San Diego, CA, USA) for 5 min. Digested cells were counted by Cellometer (Nexcelom Bioscience, Lawrence, MA, USA), plated (at the density of 2 × 10^6^ cells/well) on the Matrigel-coated six-well plate, and cultured with fresh mTeSR1 medium supplemented with 10 µM Rho-associated protein kinase (ROCK) inhibitor Y-27632 (Selleck Chemicals, Houston, TX, USA) in a hypoxic incubator. In the following day, the medium was completely replaced with fresh mTeSR1. We defined this time point as day -2. The culture medium was changed daily.

On day 0, iPSCs usually reached over 95% confluence. CM differentiation was initiated by culturing them with Roswell Park Memorial Institute (RPMI) medium (Thermo Fisher Scientific)/B-27 Supplement Minus Insulin (Thermo Fisher Scientific)/12 µM CHIR- 99021 [(glycogen synthase kinase 3 (GSK-3) inhibitor, Selleck Chemicals)] and 1% penicillin/streptomycin (Thermo Fisher Scientific). The cells were then transferred into a normoxic incubator (20% O_2_, 5% CO_2_) and cultured. Twenty-three hours after administration of CHIR, the medium was replaced with RPMI/B27 Minus Insulin and changed daily. At day 3, the medium was replaced by fresh RPMI/B27 Minus Insulin/5 µM IWP4 (Wnt/beta-catenin inhibitor, Stemgent, Lexington, MA, USA) for 48 h. At day 5, the medium was replaced with RPMI/B27 Minus Insulin and changed every two days. The RPMI medium supplemented with B27 Supplement (with insulin) (Thermo Fisher Scientific) (called control medium; Table 1) was continually used for cell culture from day 6. We examined the culture daily under the microscope and found that the cells started to beat spontaneously as early as day 8.

### 2.3. Digestion of iPSC-CMs

On day 15, spontaneously contracting cells were digested and plated onto new Matrigel-coated six-well plates. Briefly, cells were digested with 0.25% Trypsin-ethylenediaminetetraacetic acid (EDTA) (Thermo Fisher Scientific) supplemented with 5% chicken serum (Thermo Fisher Scientific) for 10–15 min at 37 °C. DMEM/F-12 with 10% fetal bovine serum (FBS, Thermo Fisher Scientific) was added to terminate the digestion. The digested cells were centrifuged (154× *g*, 3 min) and the cell pellets were resuspended with RPMI/B27 supplemented with 10% FBS and 10 µM ROCK inhibitor, and plated onto new Matrigel-coated six-well plates at the split ratio of 1:6. The following day, the medium was replaced with fresh RPMI/B27.

### 2.4. Purification of iPSC-CMs

The cell culture from CM differentiation not only included CMs but also various other cell types such as endothelial cells, fibroblasts, and undifferentiated stem cells. iPSC-CMs at day 20 were purified by culturing cells with lactate CM purification medium for 7 days to eliminate cells that are not CMs as described previously [15]. This approach is based on the remarkable biochemical differences in lactate and glucose metabolism between CMs and non-CMs. Non-CMs (e.g., endothelial cells) use glucose as their main energy source. However, CMs are capable of energy production from different sources such as lactate or fatty acids [27]. The CM purification medium used was glucose free DMEM (Thermo Fisher Scientific) supplemented with 4 mM Sodium L-lactate (Sigma-Aldrich, St. Louis, MO, USA) and 1% penicillin/streptomycin (Table 1) described previously [15]. The purity was assessed using immunofluorescence staining of cardiac troponin T (a CM-specific marker) as described below. The percentage of cardiac troponin T-positive cells was calculated by the ratio of troponin T-positive CMs/Hoechst 33342-positive total cells (representing cell nuclei) on eight different areas taken randomly per sample slide.

### 2.5. Maturation of iPSC-CMs

Purified iPSC-CMs were digested as described in “Digestion of iPSC-CMs” section and cultured in maturation medium for 3 to 7 days. The maturation medium contained glucose free DMEM (Thermo Fisher Scientific), 10 mM 4-(2-hydroxyethyl)-1-piperazineethanesulfonic acid) HEPES (Thermo Fisher Scientific), 2 mM l-carnitine inner salt (Sigma-Aldrich), 5 mM creatine (Sigma-Aldrich), 5 mM taurine (Sigma-Aldrich), 1 mM nonessential amino acid (Thermo Fisher Scientific), insulin-transferrin-selenium (Thermo Fisher Scientific), linoleic-oleic acid (Sigma-Aldrich), and 1% penicillin/streptomycin (Table 1). iPSC-CMs cultured in maturation medium for 3 to 7 days were used for analysis of bioenergetics and immunostaining, reverse transcription-quantitative polymerase chain reaction (RT-qPCR), and Western blot, respectively, as described below.

### 2.6. Immunofluorescence Staining

iPSC or iPSC-CMs were cultured on the Matrigel-coated coverslips. Cells were sequentially fixed with 4% paraformaldehyde (Electron Microscopy Sciences, Hatfield, PA, USA) for 15 min at room temperature, permeabilized with 0.5% Triton X-100 (Sigma-Aldrich) for 15 min, and blocked with 10% donkey serum (Millipore, Billerica, MC, USA) for 20 min at room temperature as described previously [19,28,29]. The cells were then stained with the following primary antibodies at 4 °C overnight: 1) mouse anti-stage-specific embryonic antigen 4 (SSEA4; 1:200 dilution, Abcam, Cambridge, MA, USA) and rabbit anti-octamer-binding transcription factor 4 (OCT4) (1:200 dilution, Abcam)(pluripotent stem cell marker), and 2) mouse anti-cardiac troponin T (1:200 dilution; Thermo Fisher Scientific, Waltham, MA, USA) and mouse anti-sarcomeric alpha actinin (1:200 dilution; Thermo Fisher Scientific) (cardiomyocyte-specific marker). After two washings with phosphate-buffered saline (PBS), the cells were incubated in the dark for one hour at 37 °C with a secondary antibody; Alexa Fluor 488 goat anti-rabbit immunoglobulin G (IgG) (1:1000, Thermo Fisher Scientific), Alexa Fluor 488 goat anti-mouse IgG (1:1000, Thermo Fisher Scientific), Alexa Fluor 594 donkey anti-mouse IgG (1:1000, Thermo Fisher Scientific), or Alexa Fluor 594 donkey anti-rabbit IgG (1:1000, Thermo Fisher Scientific). NucBlue Fixed Cell ReadyProbes Reagent (Thermo Fisher Scientific) was used to stain nuclei and mount the coverslips onto the slides. Cells were observed under a laser-scanning confocal microscope (Nikon Eclipse TE2000-U, C1 Plus).

### 2.7. Morphological Analysis

Following immunostaining and confocal microscopy imaging of iPSC-CMs, each cell area, parameter, circularity index, and cell elongation were analyzed using ImageJ (National Institutes of Health, Bethesda, MD, USA). Cell area was computed in pixels and converted to μm^2^. Cell circularity is equal to *4π × Area × Perimeter^−2^*. Elongation is the ratio of major-axis length to minor-axis length.

### 2.8. Transmission Electron Microscopy

iPSC-CMs were cultured on Matrigel-coated plastic coverslips with either control or maturation medium for 7 days. The cells were then fixed at 4 °C with 2% glutaraldehyde in 0.1 M sodium cacodylate buffer and post-fixed for one hour on ice with 1% osmium tetroxide. The cells were rinsed with distilled water and dehydrated using acetonitrile and graded methanol (50% for 20 min; 70% for 20 min; 95% for 20 min; and 100% three times for 20 min each rinse). The cells were then embedded in epoxy resin (EMbed-812, Electron Microscopy Sciences, Hatfield, PA) and polymerized at 70 °C overnight. Thin (60 nm) sections were cut and the sections were stained with lead citrate and uranyl acetate. The samples were imaged using a Hitachi H600 Electron Microscope.

### 2.9. RNA Isolation and RT-qPCR

Total RNA was extracted with the QIAzol lysis reagent (Qiagen Inc., Valencia, CA) as described previously [30]. The quantity and purity of RNA were validated using a Nanodrop spectrophotometer (Thermo Fisher Scientific). All RNA samples used were treated with DNAase (ThermoFisher Technoloiges) to remove potential DNA contamination. Complementary DNA (cDNA) was synthesized by the RT^2^ First Strand Kit (QIAGEN). For the PCR assay, cDNA was mixed with QuantiTect SYBR (a green fluorescent cyanine dye that has high affinity for double-stranded DNA) Green PCR Master Mix (QIAGEN), primers, and RNase-Free Water (QIAGEN). PCR was performed on the iCycler instrument (Bio-Rad, Hercules, CA, USA) for 10 min at 95 °C followed by 40 cycles (95 °C for 20 s, 60 °C for 30 s, and 72 °C for 30 s). All PCR reactions were performed in triplicate. The mean cycle threshold (Ct) values of triplicate wells for each sample were collected and the expression data was normalized to the endogenous control glyceraldehyde 3-phosphate dehydrogenase (GAPDH) and assessed using the comparative ΔC_t_ method. All primers were purchased from Thermo Fisher Scientific and the sequences of the primers are listed below (Table 2).

### 2.10. Protein Quantification

Protein quantification for bioenergetics analysis was conducted using the DC^TM^ Protein Assay kit (Bio-Rad) according to the manufacturer’s instructions. The absorbance at 750 nm of each sample and known standards of bovine serum albumin (BSA) was analyzed using a Microplate Reader (Bio Tek). The standard curve of absorbance vs. concentration of known standards of BSA was plotted and then used for determining the concentration of unknown proteins based on their absorbance.

### 2.11. Western Blot

iPSC-CMs were lysed on ice with radioimmunoprecipitation assay RIPA lysis buffer (Cell Signaling Technology, Danvers, MA, USA) in the presence of phenylmethylsulfonyl fluoride (Sigma-Aldrich) and phosphatase inhibitor tablets (Roche, Mannheim, Germany). The protein samples were boiled for 5 min at 97 °C. The total protein of 15 μg was loaded for Western blot assay. Blots were incubated with primary antibodies rabbit anti-myosin light chain 2 [MLC-2v; also called myosin light chain 2 (MYL2)] (Proteintech, Rosemont, IL, USA), mouse anti-myosin heavy chain 7 (MYH7, Sigma-Aldrich), or rabbit anti-GAPDH (Cell Signaling Technology) overnight on a rocker at 4 °C. The primary antibodies were then washed out with Tris-buffered saline including 0.1% Tween 20. Subsequently, the membranes were incubated with secondary antibodies conjugated to horseradish peroxidase (Cell Signaling) for one hour at room temperature. The labeled proteins were detected with ECL Prime Western Blotting Reagents (GE Healthcare, Chicago, IL, USA) and imaged using a ChemiDoc MP imaging system (Bio-Rad, Hercules, CA, USA) as previously described [31,32]. Optical densities of the proteins were quantified. The MYL2 and MYH7 abundance in matured iPSC-CMs was normalized to GAPDH and presented as a percentage of the non-maturation control iPSC-CMs.

### 2.12. Mitochondrial Bioenergetic Analysis

Purified iPSC-CMs were seeded onto Matrigel-coated Seahorse 96 assay plates at a density of 10,000 cells/well and allowed to grow for 3 days with normal culture medium. The bioenergetic assay was carried out 3 days after culturing with either control or maturation medium. Mitochondrial oxidation and glycolysis were evaluated by analysis of oxygen consumption rate (OCR, pmol/min/µg protein) and extracellular acidification rate (ECAR, mpH/min/µg of protein) using a XF96 Extracellular Flux Analyzer (Agilent, Santa Clara, CA, USA), respectively. One hour prior to the assay, the culture medium was changed with assay medium contained unbuffered RPMI supplemented with 100 nM insulin (Sigma-Aldrich) and 11.1 mM glucose (for mitochondrial oxidation assay) or no glucose (for glycolysis assay). For the mitochondrial oxidation assay, OCRs were obtained from the slope of change in oxygen over time. After measurements of the baseline OCR, OCRs were analyzed by sequential automatic injections of the following substrates and inhibitors with a final concentration of 10 μM oligomycin (adenosine triphosphate (ATP) synthase inhibitor) (Sigma-Aldrich), 2 μM carbonyl cyanide *p*-(trifluoromethoxy) phenylhydrazone (FCCP, uncoupler of oxidative phosphorylation in mitochondria) (Sigma-Aldrich), and 10 μM antimycin A (electron transport chain complex III blocker) (Sigma-Aldrich).

Seahorse XF Palmitate-BSA FAO (fatty acid β-oxidation) Substrate kit (Agilent, Santa Clara, CA, USA) was used to assess the mitochondrial oxidation capacity of matured and non-matured iPSC-CMs in response to the exogenous fatty acid (palmitate). iPSC-CMs were pretreated with the following reagents: (1) 200 μM palmitate conjugated to 33 μM bovine serum albumin (BSA, Agilent), 33 μM BSA as a solvent control, 100 μM etomoxir (ETO, a specific irreversible inhibitor of carnitine palmitoyltransferase 1, Agilent), or 25 mM 2-deoxy-D-glucose (2-DG, competitive glycolytic inhibitor) (Sigma-Aldrich) for 30 min prior to the mitochondrial oxidation assay according to the assay group. Each parameter for OCR we assessed was calculated as described below: (1) basal respiration: The last rate measurement before oligomycin injection minus the non-mitochondrial respiration rate (the minimum rate measurement after antimycin A injection); (2) ATP production: the last rate measurement before oligomycin injection minus the minimum rate measurement after oligomycin injection; (3) maximal respiration: the maximum rate measurement after FCCP injection minus non-mitochondrial respiration rate; (4) spare respiratory capacity: the maximum respiration minus basal respiration; (5) basal respiration due to utilization of exogenous fatty acids (palmitate): basal palmitate-BSA/ETO (minus) rate minus basal BSA/ETO (minus) rate; (6) maximal respiration due to utilization of exogenous fatty acids: maximal palmitate-BSA/ETO (minus) rate minus maximal BSA/ETO (minus) rate. All values of OCR parameters calculated were normalized to the quantified protein content.

### 2.13. Glycolysis Analysis

For the glycolysis assay, ECARs were obtained from the slope of change in H^+^ concentration over time. After measurements of the baseline ECAR, ECARs were analyzed by sequential automatic injections of the following substrates and inhibitors with a final concentration of 5.5 μM glucose, 10 μM oligomycin, and 25 mM 2-DG. Glucose in the concentration of either 1, 3, 5.5, or 11.1 mM was injected to analyze the effect of different glucose concentrations on the ECAR of matured and non-matured iPSC-CMs. Each assessed parameter for ECAR was calculated as: (1) glycolysis: the maximum rate measurement before oligomycin injection minus the last rate measurement before glucose injection, (2) glycolytic capacity: the maximum rate measurement after oligomycin injection minus the last rate measurement before glucose injection, and (3) glycolytic reserve: the glycolytic capacity minus glycolysis. All calculated values of ECAR parameters were normalized to the protein content.

### 2.14. Statistical Analysis

Data was obtained from four independent cardiomyocyte differentiations. Values presented are the means ± standard errors of the mean (SEM). Statistical analysis was conducted using GraphPad Prism version 6.0. Statistical difference was analyzed by the Student’s t-test when comparing two groups or a one-way Analysis of variance (ANOVA) with post-hoc Tukey test for multiple comparisons. *P*-values less than 0.05 were considered statistically significant.

## 3. Results

### 3.1. Characterization of iPSCs and iPSC-derived CMs

iPSCs cultured on Matrigel-coated plates formed colonies with clear boundaries (Figure 1B-a). These colonies consistently stained positive with pluripotent stem cell-specific markers OCT4 and SSEA4 (Figure 1(B-b,B-c)). iPSC-derived CMs started spontaneous contraction after 8 days of differentiation. The digested, replated, and cultured iPSC-CMs at post differentiation day 20 grew as a monolayer, showing a clear cell shape on a Matrigel-coated plate after the first digestion (Figure 1C-a). Cells around post differentiation day 20 were positively stained by CM-specific markers troponin T and sarcomeric α-actinin (Figure 1(C-b,C-c)). iPSC-CMs cultured in lactate purification medium for 7 days resulted in a significant increase in troponin T-positive cells from 75% to 98% (Figure 2A,B). We observed floating dead cells during the 7-day purification procedure (data not shown). The number of cells in the culture changed over the course of CM differentiation and purification. About 2 × 10^6^ iPSCs/well in six-well plates were plated 2 days prior to the initiation of CM differentiation. On days 15 to 20 after CM differentiation, the cell number reached around 3 to 4 × 10^6^/well. On day 27, 60 to 80% cells were left in the wells compared to day 20 due to the death of non-CMs during the lactate purification process.

### 3.2. The Effect of Maturation Medium on Morphological and Ultrastructural Change, and CM-related Gene and Protein Expression in iPSC-CMs

The confocal images show that iPSC-CMs cultured in control normal CM culture medium (control medium) were round and displayed shorter lengths (Figure 3(A-a,A-c)), while the cells treated with maturation medium for 7 days exhibited a prolonged and rod-like shape, and more organized and aligned patterns with clear sarcomeric striations (Figure 3(A-b,A-d)). Maturation medium also led to elongated CMs as evidenced by the increased cell perimeter, circularity, and elongation in maturation medium-treated iPSC-CMs (Figure 3B). Next, iPSCs-CMs treated with control or maturation medium were fixed and imaged using electron microscopy to visualize the cellular ultrastructure and mitochondrial morphology. In control non-matured iPSC-CMs, the myofibrils are plentiful but somewhat haphazardly organized within the cells. The Z-lines of the muscle fibers are visible although mostly misaligned with those of adjacent fibers. There are occasional structures that resemble intercalated disks. A few mitochondria are seen within the cell cytoplasm or associated with the myofibrils. In matured iPSC-CMs, the myofibrils are plentiful and well organized in parallel rows. The Z-lines of the muscle fibers are visible and mostly aligned with those of adjacent fibers. There are occasional structures that resemble intercalated disks. Numerous mitochondria are seen within the cell cytoplasm and associated with the myofibrils (Figure 3C).

We also analyzed the effect of a 7-day maturation medium treatment on the CM maturation-related gene expression profiles of iPSC-CMs (Figure 3D). In agreement with the structural maturation as described above, most mature CM-related sarcomere protein encoding genes [(e.g., troponin (TNN)T2, TNNI3, myosin light chain (MYL)2, MYL3, MYL4, myosin heavy chain (MYH)6 and myosin binding protein C (MYBPC3) were significantly increased in maturation medium-treated iPSC-CMs, but the expression levels of TNNI1, MYL7 and MYH7 were not influenced. We also observed that maturation medium treatment induced the upregulation of several mature CM-related genes such as 1) ion channel genes sodium voltage-gated channel alpha subunit 5 (SCN5A), potassium voltage-gated channel subfamily J (KCNJ)4, KCNJ12] and 2) the genes of encoding sarcomeric calcium handling-related protein cardiac ryanodine receptor (RYR2) and transcriptional regulator peroxisome proliferator-activator receptor alpha (PPARα) (Figure 3D). PPARα is related to fatty acid metabolism in adult CMs [20,21,22]. MYL2 and MYH7 protein expression levels were consistent with their gene expression data (Figure 3E and Figure 7A). These data suggest that the maturation medium promotes iPSC-CMs towards the maturation process in multiple aspects including cell morphology, sarcomeric structure, ultrastructural change, and gene and protein expression.

### 3.3. The Effect of Maturation Medium on the Mitochondrial Oxidation of iPSC-CMs

As CMs mature, the mitochondrial oxidative capacity increases. We then investigated the effect of maturation medium on mitochondrial respiration function by the sequential addition of oligomycin, FCCP and antimycin A (Figure 4A). We observed that the maturation medium significantly increased the OCRs that were linked to basal respiration, ATP production, maximal respiration and spare respiratory capacity (Figure 4B,C). The increased values of these major mitochondrial oxidation parameters indicate the maturity or enhanced mitochondrial oxidative capacity.

### 3.4. The Effect Of Exogenous Fatty Acids (Palmitate) on the OCR of iPSC-CMs

Glycolysis is a major energy source of immature CMs while FAO is a major energy source in adult CMs [23,24]. We pretreated iPSC-CMs with both palmitate (or BSA) and ETO and then measured oxidation of exogenous (palmitate) fatty acid oxidation. We found that non-matured iPSC-CMs displayed minimal changes in basal OCR and a modest increase in maximum OCR in response to the palmitate treatment. The matured iPSC-CMs displayed a larger change in both basal and maximal respirations than non-matured control iPSC-CMs when the exogenous palmitate was added (Figure 5A). Pretreatment with ETO but not 2-DG abolished the palmitate-mediated OCR increases in maturation medium-treated iPSC-CMs (Figure 5B,C), suggesting that the palmitate-mediated increased OCR resulted from fatty acid oxidation but not glycolysis, and these matured iPSC-CMs have a stronger ability to utilize fatty acids as energy substrates for ATP production as observed in adult CMs.

### 3.5. The Effect of Maturation Medium on the Glycolysis of iPSC-CMs

We measured the glycolytic capacity based on ECAR by the sequential additions of glucose, oligomycin and 2-DG. The result showed that the ECAR level in glycolysis, glycolytic capacity and glycolytic reserve were significantly higher in maturation medium-treated iPSC-CMs ( Figure 6(A-b,A-c) and Figure 7B-b). The glucose-induced glycolysis increases observed suggests an enhanced glycolytic process after glucose starvation in matured iPSC-CMs compared to non-matured iPSC-CMs. Enhanced glycolytic capacity indicates an increased cellular glycolytic response in the matured iPSC-CMs to energetic demand from stress. The increase of glycolytic reserve indicates the enhanced capacity available to utilize glycolysis beyond the basal rate in the matured iPSC-CMs. Together, the increased values of these three key glycolytic flux parameters are associated with the glycolytic capacity and maturity in the maturation medium-treated iPSC-CMs. Furthermore, the glycolysis, glycolytic capacity, and glycolytic reserve characteristics exhibited the greater response to the glucose in matured iPSC-CMs in a dose-dependent manner (Figure 6C) than in non-matured iPSC-CMs (Figure 6B). A possible explanation for the higher ECAR could be that maturation medium-treated iPSC-CMs have more capacity to switch the energetic pathway from fatty acid β-oxidation to glycolysis when the fatty acid source is compromised.

Taken together, iPSC-CMs cultured in maturation medium have higher mitochondrial oxidative capacity, stronger ability of utilizing fatty acids as energy sources, and higher adaptive ability to alter the energy substrate against environmental (e.g., energy demand, substrate, and stress) changes compared to control medium-treated iPSC-CMs, resembling adult CM-like characteristics. The maturation medium-induced higher mitochondrial oxidative capacity and glycolytic capacity were consistently observed in two different iPSC lines (Figure 4, Figure 6 and Figure 7).

## 4. Discussion

Recently, the immature properties of human iPSC-CMs have attracted great attention. In this study, we took advantage of the adult CMs’ unique major energy substrate utilization property to mature iPSC-CMs by culturing them in fatty acid-contained maturation medium as described previously [26]. We then further investigated whether fatty acid utilization could enhance the maturation of iPSC-CMs in cell structure, morphology, gene expression, and energy metabolism. The results showed that fatty acid-contained medium enhanced iPSC-CM maturation in several aspects such as cell morphology, structure, gene and protein expression, and metabolism.

In order to study the maturation of iPSC-CMs, it is essential to have an effective CM differentiation and purification protocols. Several such differentiation protocols have been developed, such as using recombinant factors (e.g., bone morphogenetic protein 4) toward chemical compounds for directing CM induction [33,34,35], through either a two-dimensional culture or a suspension culture system [19,36]. Additionally, there have been several methods developed for the purification of CMs from heterogeneous populations such as surface-based enrichment using fluorescent activated cell sorting and lactate-based metabolic enrichment [27]. The development of purification technology represents major progress in the field and provides unprecedented opportunities for iPSC-CM-based therapy, disease modeling, and drug testing. One recent study from Ban et al. demonstrated a CM differentiation platform enabling the efficient production of iPSC-CMs in suspension culture. The process robustness and efficiency was ensured by CHIR- and IWP4-based uninterrupted Wnt pathway control at early stages of differentiation and resulted in >90% CMs from iPSCs [36].

In order to better utilize iPSC-CMs and avoid misinterpretation of the results from non-CMs contained in the differentiation culture, we obtained a robust and efficient cardiac differentiation. We used the 2D monolayer-based directed CM differentiation protocol from Lian et al. [18] with minor modifications, on undifferentiated healthy iPSCs. Our protocol was developed for the generation of CMs by temporal modulation of Wnt signaling on iPSCs through sequential treatment with a GSK-3 inhibitor (CHIR) and Wnt inhibitor (IWP4). We observed high CM differentiation efficiency. We also used lactate medium to obtain the purified CMs. The successful rate of cardiac differentiation and purification was high with almost 100% reproducibility in these two iPSC lines as evidenced by the spontaneous cell contraction and the expression of cardiac-specific protein (troponin T) on 98% cells in the culture (Figure 2).

The maturation of CMs is complex and it is difficult to pinpoint a specific maturation marker. Maturation may be defined by a collective set of changes observed in cell morphology, cellular structure, gene expression level, electrophysiology, contractility, and metabolism [37,38]. In this study, we focused on the evaluation of cell morphology, structure, gene and protein expression level, and metabolic aspects in iPSC-CMs. iPSC-CMs are significantly immature compared to adult CMs and exhibit a fetal-like phenotype such as cell morphology. When comparing iPSC-CMs to the developmental CM stages, there exhibits apparent morphological similarities to early fetal CM stages. iPSC-CMs and fetal CMs are morphologically round. CMs switch to an elongated morphology over maturation. Mature adult CMs are elongated and rod-shaped [38]. We found that maturation medium promoted elongation of iPSC-CMs (Figure 3B). A sarcomere is the contractile unit in CMs, which consists of proteins forming thick and thin filaments. The sarcomeres in adult CM are highly aligned and contain high densities of well-organized myofibrils [37,38] while iPSC-CMs have an undefined orientation with disorganized myofibrils (Figure 1C and Figure 3A,C). The expression levels of protein assembled in the sarcomere change during CM maturation. For example, fetal and neonatal CMs express mostly skeletal troponin I (TNNI1) which is replaced by higher amounts of cardiac troponin I (TNNI3) during CM development. TNNI3 is mostly found in adult CMs [39]. We found that cardiac troponin T (TNNT2)-positively stained sarcomere striations are more organized in iPSC-CMs treated with maturation medium (Figure 3). Consistently, several mature CM-related sarcomere protein gene expressions (e.g., TNNT2, TNNI3, MYL2, MYL3, MYL4, MYH6 and MYBPC3) were also upregulated in these cells. Our observations are consistent with a previous report [26].

Action potentials are unique for each CM and govern CM electrical behavior which is critical for heart function. Most adult CMs, except for pacemaker cells are quiescent and will contract upon receiving stimulation from pacemaker cells while immature CMs exhibit spontaneous contraction due to the availability of different ion channels on the cells. It was reported that fatty acids enhanced sodium current of iPSC-CMs and increased action potential upstroke velocity over the maturation process [26]. Consistently, we found that the expression of several mature CM-related ion channels such as potassium channel α-subunit (KCNJ4 and KCNJ12) and voltage-dependent sodium channel subunit (SCN5A) were increased in iPSC-CMs treated with maturation medium (Figure 3D). Taken as whole, our observations combined with the previous report of Drawnel et al. [26] suggest that the maturation medium promotes iPSC-CMs toward the maturation process in sarcomere organization, molecular, and electrophysiological aspects.

Energy metabolism is the process of generating energy of ATP from nutrients. Different cell types have their own distinctive metabolic profiles and preference of utilizing different energy substrates (e.g., glucose, fatty acids, and lactate) in correspondence with their functional activity. Metabolic energy demand in the beating heart is high at rest compared with other tissues and increases dramatically during physical activity. As mentioned in the Introduction section, during early cardiac development, glycolysis is a major source of energy. As CMs mature and become terminally differentiated, mitochondrial oxidative capacity increases. Our data showed that maturation medium significantly increased the oxygen consumption rate linked to the basal respiration, ATP production, and maximal respiration and spare capacity (Figure 4), mimicking the enhanced mitochondrial oxidative capacity in matured iPSC-CMs. These observations are consistent with the recent reports of similar approaches [40,41]. The increased oxidative capacity might be associated with the increased mitochondrial numbers, improved mitochondrial morphology (e.g., enhanced cristae development), and/or electron transport chain activity. Cristae are folds that give mitochondria a significant surface area for efficient cellular respiration. They are densely packed within adult CMs but absent or poorly developed within fetal and iPSC-CMs, leading to the decreased mitochondrial function [42,43]. In agreement with these assumptions, the electron microscope images revealed that maturation medium-treated iPSC-CMs showed well organized plentiful myofibrils with aligned visible Z-lines and more mitochondria within the cells compared with the cells cultured in control medium.

Immature fetal CMs and iPSC-CMs use glucose mostly as an energy source via glycolysis, which is less efficient compared with fatty acid β-oxidation [44]. Glycolysis is the major pathway of glucose metabolism and can occur aerobically or anaerobically depending on whether oxygen is available. Under aerobic conditions, 32 mol ATP/mol glucose is generated, and under anaerobic conditions, only 2 mol ATP/mol glucose is generated. On the other hand, complete fatty acid β-oxidation generates 106 mol ATP/mol palmitate [22,42,43]. Fatty acids have a higher efficiency in ATP production compared to glucose metabolism. Many efforts partially elucidated the mechanisms of the metabolic changes from aerobic glycolysis to fatty acid β-oxidation in immature fetal CMs and iPSC-CMs. For example, a recent study reported that the inhibition of the pathway of HIF1α (hypoxia-inducible factor 1α) and LDHA (lactate dehydrogenase A) resulted in metabolic shift from aerobic glycolysis to oxidative phosphorylation, leading the improvements of metabolic and functional maturation of iPSC-CMs [45]. We observed a greater OCR elevation in matured iPSC-CMs than in non-matured cells in response to exogenous fatty acids (palmitate) (Figure 5A). ETO (an irreversible inhibitor of CPT1) but not 2-DG (an inhibitor of glycolysis) abolished the palmitate pretreatment-mediated OCR increases in matured iPSC-CMs, suggesting that these matured iPSC-CMs have a strong capacity of utilizing fatty acids as energy substrates for ATP production as observed in adult CMs. Additionally, we observed that matured iPSC-CMs exhibit a stronger glycolytic capacity compared to immature iPSC-CMs (Figure 6), suggesting that matured iPSC-CMs have a higher capacity to switch to glucose utilization when fatty acid oxidation is compromised. These data are in line with the previous observation that adult CMs retain capability to readily switch to glucose or other substrate utilization when fatty acids are not available or fatty acid oxidation is compromised [46].

Although the results of the inhibitor studies support that fatty acids promoted the maturation of iPSC-CMs as discussed above, we cannot exclude the potential contribution of other components (e.g., taurine, carnitine, creatine, and insulin-transferrin-selenium from B7 solution) in the maturation medium to the maturation of iPSC-CMs. Taurine plays an important role in the absorption of fat and is utilized not as a nutritious essential amino acid but as one of the “conditionally essential’’ amino acid in infant nutrition [47]. Taurine is also considered to act as a pH buffer in the mitochondrial matrix to stabilize beta-oxidation of fatty acids [48]. Carnitine helps to transport fatty acids through the mitochondrial membrane and is essential for fatty acid beta-oxidation [48]. Selenium is an essential trace element for humans and is also known as an essential factor for cell culture when a serum-free medium is used together with insulin and transferrin. Without selenium, cells can neither proliferate nor survive [49]. Thus, in addition to fatty acid, other supplemental components contained in maturation medium might also involve the maturation process of iPSC-CMs through the fatty acid metabolism pathway.

In conclusion, our data showed that fatty acid-contained maturation medium can promote iPSC-CMs to undergo maturation. The matured iPSC-CMs exhibit adult CM-like metabolic phenotypes such as enhanced mitochondrial oxidative function, preference of utilizing fatty acids as energy substrates, and a strong ability to switch to use glucose as energy sources when needed. Mitochondrial metabolic profiles have been reported to be associated with stem cell fate, cell proliferation, and differentiation [50]. CM maturation is a complicated process of encompassing several development event changes (e.g., gene expression, structural, electrophysiological, and calcium handing, contractility, and mitochondrial metabolism profile changes). Enhanced mitochondrial oxygen capacity and fatty acid oxidation might promote CM maturation-related gene expression, improve sarcomere organization. Many of the mechanisms remain unknown regarding: 1) How fatty acid-mediated metabolism shifts influence the maturation of cells in other aspects, and 2) What molecular and signaling pathways are involved in the interplay between mitochondria and CM maturation. Nevertheless, the observations from this study stress the importance of assessing the effect of fatty acids on the metabolic profiles in iPSC-CMs. Understanding the basis of crosstalk between fatty acids, mitochondrial metabolism, and the maturation of iPSC-CMs is of critical importance, to have a better understanding of the mechanisms of CM maturation. In addition, the fatty acid-mediated matured iPSC-CMs with adult CM-like metabolic profiles might represent a promising human CM source in myocardial regeneration, disease modeling, and drug assessment. Finally, these matured highly purified iPSC-CMs with enhanced metabolic maturation will serve as a valuable model that allows the study of metabolic changes mediated by drugs or cardiac diseases.

## Figures and Tables

**Table 1:** The components of different cell culture medium.

Medium Name	Component	Supplier	Cetology Number	Final Concentration
iPSC culture medium	mTeSR1	STEMCELL Technologies	85850	1×
	Penicillin-Streptomycin	Thermo Fisher Scientific	15140122	100×
Control medium	RPMI 1640 Medium	Thermo Fisher Scientific	11875093	1×
	B-27 Supplement (50X), serum free	Thermo Fisher Scientific	17504044	50×
	Penicillin-Streptomycin	Thermo Fisher Scientific	15140122	100×
Lactate purification medium	DMEM, no Glucose	Thermo Fisher Scientific	11966025	1×
	Sodium L-lactate	Sigma-Aldrich	71718	4 mM
	Penicillin-Streptomycin	Thermo Fisher Scientific	15140122	100×
Maturation medium	DMEM, no Glucose	Thermo Fisher Scientific	11966025	1×
	HEPES (1M)	Thermo Fisher Scientific	15630080	10 mM
	L-carnitine inner salt	Sigma-Aldrich	C0158	2 mM
	Creatine	Sigma-Aldrich	C0780	5 mM
	Taurine	Sigma-Aldrich	T0625	5 mM
	MEM (non-essential amino acids) Solution	Thermo Fisher Scientific	11140076	100×
	Insulin, Transferrin, Selenium Solution (ITS-G)	Thermo Fisher Scientific	41400045	100×
	Linoleic Acid-Oleic Acid-albumin	Sigma-Aldrich	L9655	100×
	Penicillin-Streptomycin	Thermo Fisher Scientific	15140122	100×

Note: iPSC: induced pluripotent stem cell; s); DMEM: Dulbecco’s modified Eagle mediuM; RPMI: Roswell Park Memorial Institute; HEPES: 4-(2-hydroxyethyl)-1-piperazineethanesulfonic acid.

**Table 2:** Primer sequences for reverse transcription-quantitative polymerase chain reaction RT-qPCR).

Gene	Forward Primer Sequence (5′to 3′)	Reverse Primer Sequence (5′to 3′)	PCR Product Length (bp)
*GAPDH*	GTCTCCTCTGACTTCAACAGCG	ACCACCCTGTTGCTGTAGCCAA	131
*TNNT2*	AGCATCTATAACTTGGAGGCAGAG	TGGAGACTTTCTGGTTATCGTTG	112
*TNNI1*	CAGCTCCACGAGGACTGAAC	CTCTTCAGCAAGAGTTTGCG	101
*TNNI3*	CCTCAAGCAGGTGAAGAAGG	CAGTAGGCAGGAAGGCTCAG	134
*MYL2*	ACATCATCACCCACGGAGAAGAGA	ATTGGAACATGGCCTCTGGATGGA	247
*MYL3*	GCCCTAAGGAGGTCGAGTTT	ACACTGCCCGTAGGTGATCT	137
*MYL4*	GACTTCACTGCCGACCAGAT	CTCGGCATTGGTAGGGTTCT	90
*MYL7*	CCGTCTTCCTCACGCTCTT	TGAACTCATCCTTGTTCACCAC	120
*MYH6*	GCTGGCCCTTCAACTACAGA	CTTCTCCACCTTAGCCCTGG	106
*MYH7*	GAGGACAAGGTCAACACCCT	CGCACCTTCTTCTCTTGCTC	95
*MYBPC3*	GGCATGCTAAAGAGGCTCAA	TCTTGTGGCCTTTGCTCAC	103
*SCN5A*	ACTGCACAATGACCAGCAGGA	GTGAGAAGTGCTCGATTAGTTCAGACA	77
*KCNJ4*	CCTTGGGATGTAGCGCC	GTCCGTGCATGTCCTGAAG	109
*KCNJ12*	TGGATCCTTTCCAGTTGGTG	CGGCTCCTCTTGAGTTCTATCTT	114
*KCNH2*	CACCGCCCTGTACTTCATCT	AGGCCTTGCATACAGGTTCA	118
*KCNQ1*	CGCCTGAACCGAGTAGAAGA	TGAAGCATGTCGGTGATGAG	71
*KCND2*	CTACCTGTTCCGGTGATTGTATCC	TCTTTTGTGCCCTTCGTTTGT	82
*KCND3*	CCAATTCTAACCTGCCAGCTAC	CTGCTTTCAAATTAAGGCTGGA	122
*CACNA1C*	CAATCTCCGAAGAGGGGTTT	TCGCTTCAGACATTCCAGGT	78
*GJA1*	CAATCACTTGGCGTGACTTC	AAAGGCAGACTGCTCATCTC	211
*SERCA2A*	TTGGCAGGAGCGGAACGCAG	CAACCCGCAGCGTGGTGGAT	209
*RYR2*	AAGCCCTCTCGTCTGAAACA	CCACCCAGACATTAGCAGGT	193
*PPAR* *α*	ATTACGGAGTCCACGCGTGTG	TTGTCATACACCAGCTTGAGT	76

Note: GAPDH: glyceraldehyde 3-phosphate dehydrogenase; TNNI: troponin I; TNNT: troponin T; MYL: myosin light chain; MYH: myosin heavy chain; MYBPC: myosin binding protein C; SCN5A: sodium voltage-gated alpha subunit 5; KCNJ: potassium inwardly rectifying channel subunit J; KCNH: potassium voltage-gated channel subfamily H; KCNQ: potassium voltage-gated channel subfamily Q; KCND: potassium voltage-gated channel, subfamily D; CACNA1C: calcium voltage-gated channel subunit alpha1 C; GJA1: gap junction protein, alpha 1; SERCA2A: sarcoplasmic/endoplasmic reticulum Ca^2+^ATPase 2a; RYR2: cardiac ryanodine receptor; PPARα: peroxisome proliferator-activator receptor alpha.
